# First person – Priyanka Sarkar

**DOI:** 10.1242/bio.060184

**Published:** 2023-10-24

## Abstract

First Person is a series of interviews with the first authors of a selection of papers published in Biology Open, helping researchers promote themselves alongside their papers. Priyanka Sarkar is first author on ‘ Glucose to lactate shift reprograms CDK-dependent mitotic decisions and its communication with MAPK Sty1 in *Schizosaccharomyces pombe*’, published in BIO. Priyanka is a PhD student in the lab of Dr Geetanjali Sundaram at the University of Calcutta, Kolkata, India, investigating how metabolic alterations can affect mitotic entry decisions in *Schizosaccharomyces pombe*.


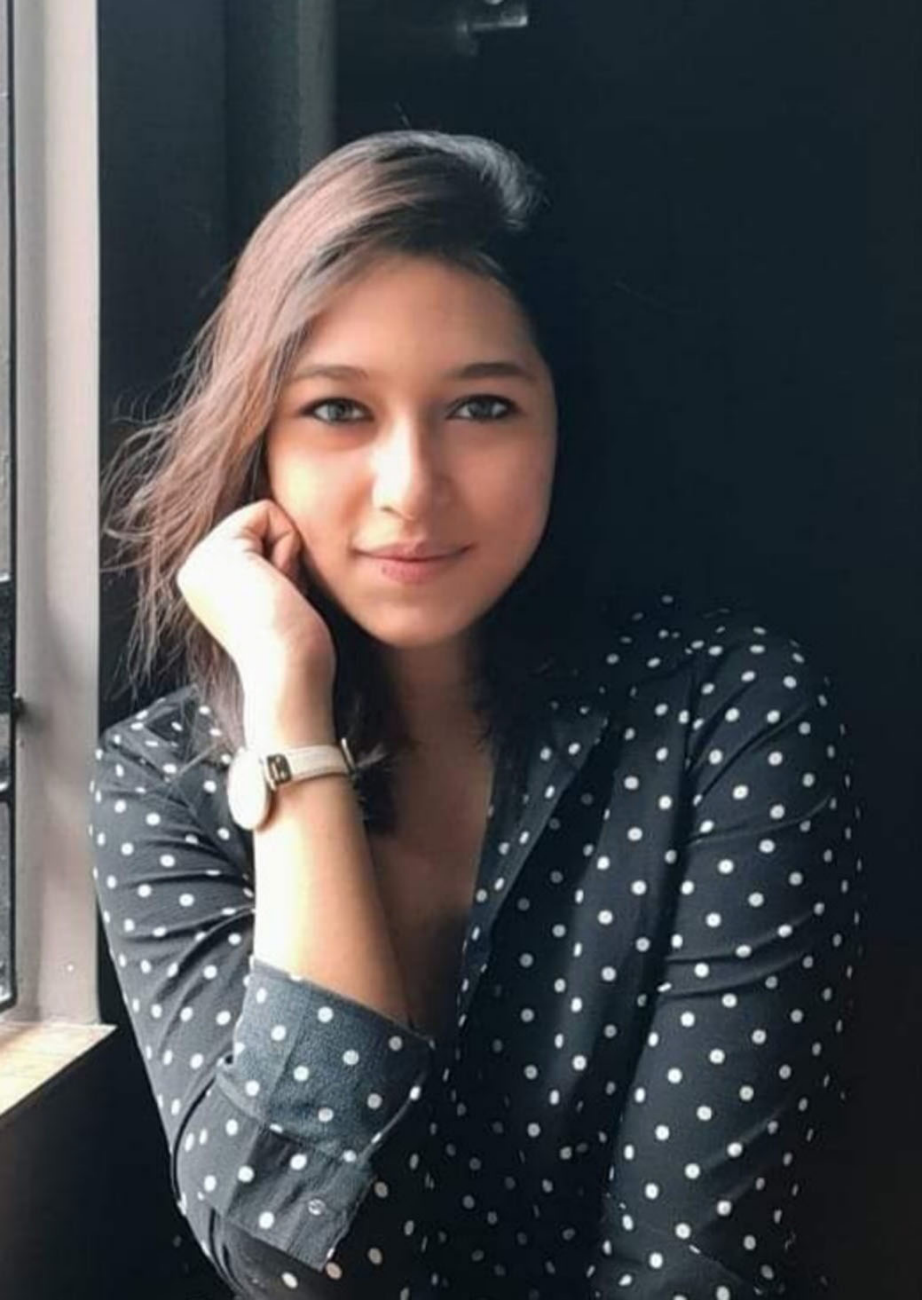


**Priyanka Sarkar**

**Describe your scientific journey and your current research focus**

My Masters’ dissertation was related to understanding how ageing affects DNA repair pathways throughout the cell cycle. Since then, I developed an interest in understanding the delicate control mechanisms that make the cell cycle a successful event. Later on, I questioned as to how physiological stressors affect mitotic decisions, which provoked me to work with cyclin dependent kinase (CDK) mutants and how they behave in response to stressors. Currently, I'm intrigued by how just altering carbon sources in growth media can bring about completely opposite outcomes when deciding on whether to commit to mitosis or to halt it at a checkpoint. I wish to further look into the exact mechanisms that dictate such decisions for which cells behave so differently upon altering the carbon sources.

**Who or what inspired you to become a scientist?**

My grandfather, the late Dr Ajit K. Maiti, who was awarded the Shanti Swarup Bhatnagar Prize for Science and technology in the year 1971, inspired me in my early years to pursue research. He had done pioneering work aimed at understanding the autonomic and viscero-vegetative functions of spinal cord physiology. As a child, I saw him have this immense power to comprehend basic mechanisms that regulate human processes and this always made me think that research probably is next to having a super power, which, if exercised in the right direction, can bring you to a table of secrets, those that are your sole findings. The glimpses to the bigger truth are what I feel is a researcher's pride and I wished to embark on this journey.

**How would you explain the main finding of your paper?**

A basic process linked to many diseases is cell cycle regulation in response to biochemical stimuli. It is unclear how such reactions are regulated in a complicated metabolic context. This study exposes hitherto unrecognised facets of *Schizosaccharomyces pombe*'s metabolic regulation of cell division. We demonstrate that switching the carbon supply from glucose to lactic acid modifies the activities of the mitogen-activated protein kinase (MAPK) Spc1 and CDK Cdc2, resulting in unexpected changes in the behaviour and fate of such cells. Our findings indicate that there is cross-talk between Cdc2 and Spc1, which compromises the Spc1-dependent control of Cdc2 activity. In addition, the presence of lactate also causes a reversal in the link between Cdc2 activity and mitotic time. We further demonstrate that the altered molecular functions indicated above as well as the altered behaviour of these cells are significantly influenced by the biochemical status of cells under these settings.

**What are the potential implications of this finding for your field of research?**

CDKs serve crucial roles in the control of cell cycle transition and are strongly linked to the emergence of malignancies. CDK activity is governed by an intricate regulatory network of genetic and epigenetic pathways, but, as cancer progresses, this network becomes dysregulated. Our research suggests that there is still a great deal to learn about the biochemical elements that can control CDK function. As revealed in this study, more investigation into the function and underlying processes of CDKs in the presence of complex metabolic cues will pave the way for improved CDK-targeting cancer treatments in the future.

**Figure:**
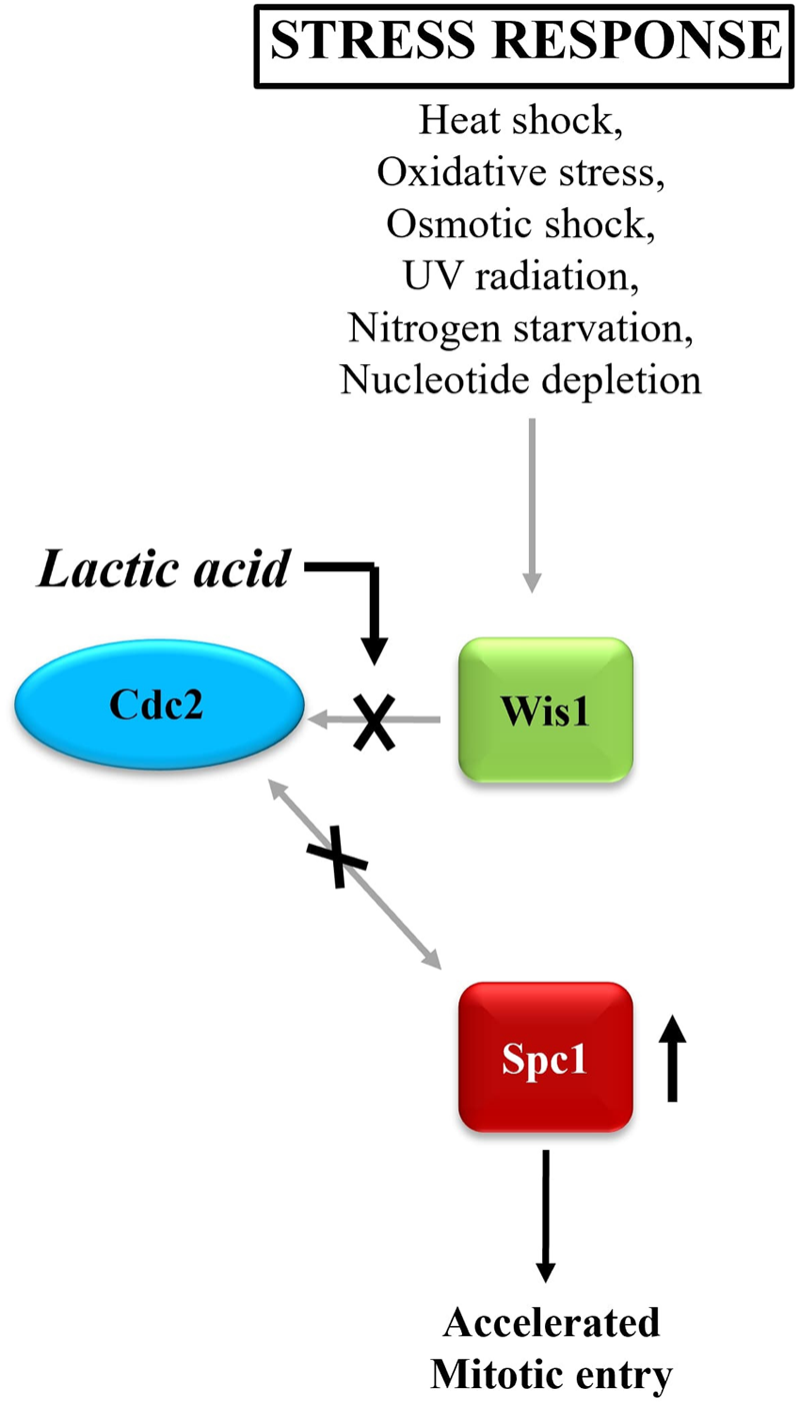
The known correlation between Cdc2 activity and levels of phosphorylated Spc1 is altered when cells are grown in lactic acid as an altered source of carbon.

**Which part of this research project was the most rewarding?**

It is widely known that neoplastic cells utilise the beneficial properties of the byproduct lactate. However, how lactate affects cell division control mechanisms at the mitotic level and how some mutations help with better utilisation of the other supply of carbon, despite glucose being the preferred source, were unknown. During the course of my PhD, I came to the startling realisation that the relationship between cellular metabolism and the cell cycle machinery is bidirectional. I'd love to delve more and gradually look for explanations for the phenotypic or molecular alterations I described in this piece.

**What do you enjoy most about being an early-career researcher?**

The most reassuring aspect of being a young researcher is that I have time to look for answers to my questions, and also the fact that I'll meet people who must have had similar questions to ask and get to know an alternative point of view, which might help me narrow my search for solutions.

**What piece of advice would you give to the next generation of researchers?**

Be patient and have faith in what you believe.

**What's next for you?**

I'd love to pursue research, complete my PhD and look for suitable laboratories that align to my research questions, until I build a lab of my own.
